# Virological outcomes in ARV-naïve patients switching or not from a first successful boosted PI-regimen to efavirenz, nevirapine or abacavir regimens

**DOI:** 10.1186/1758-2652-13-S4-O21

**Published:** 2010-11-08

**Authors:** T Bommenel, JL Meynard, O Launay, A Simon, A Mahamat, V Martinez, J Gilquin, C Katlama, AS Lascaux, C Pradier, E Rouveix, D Costagliola, S Abgrall

**Affiliations:** 1 INSERM U 943, BP 335, 56 boulevard Vincent Auriol, Paris, France; 2 AP-HP, Hôpital Saint-Antoine, Paris, France; 3 AP-HP, Hôpital Cochin, Paris, France; 4 AP-HP, Groupe hospitalier Pitié-Salpétrière, Paris, France; 5 Centre Hospitalier Andrée Rosemon, Cayenne, France; 6 AP-HP, Hôpital Antoine Béclère, Clamart, France; 7 AP-HP, Hôpital Hotel-Dieu, Paris, France; 8 AP-HP, Hôpital Henri-Mondor, Creteil, France; 9 Hôpital de l’Archet 1, Nice, France; 10 Hôpital Ambroise Paré, Boulogne, France; 11 INSERM U 943, Paris, France

## Objectives

To compare virological outcomes in patients who switched to a cART including efavirenz (EFV), nevirapine (NVP) or abacavir (ABC) with patients who continued on a first virologically successful boosted protease inhibitor (PI)-containing cART, and to assess virological differences between the switch regimens.

## Methods

Using the French Hospital Database on HIV (FHDH-ANRS Co4), 439 antiretroviral (ARV)-naive patients with undetectable viral load (VL) who switched from a first boosted PI-containing cART to a combination including EFV (n=196), NVP (n=123) or ABC (n=120) were selected. Each patient was matched with 3 patients who did not change their cART on the basis of sex, age, CD4 cell count, VL and date of the first cART initiation and duration of undetectability. Time to virological failure (VF) was analysed using Kaplan-Meier curves and Cox models. Potential confounding variables considered for the analyses were HIV transmission group, at the date of first PI-cART initiation: NRTI backbone, PI drug; at the index date: AIDS status, CD4 cell count, NRTIs background, calendar period, time since inclusion in the database, time since first PI-cART initiation, time between undetectability and switch. Each variable associated with VF in the univariate model (p<0.20) was included in a multivariable model designed to evaluate the impact of the sole switch first, then the impact of the switch regimen, on the risk of VF. Each model was stratified by the matched groups (exposed/matched non-exposed patients).

## Results

12-month probabilities of VF were 3.7% in patients not switching and 5.7% in patients switching, 3.9%, 7.2% and 9.0% in patients switching to EFV-, NVP- and ABC-cART, respectively. After adjustment on PI at first cART, CD4 cell counts and AIDS status at the date of switch, switch was not associated with VF (crude HR, 1.20; 95%CI,0.81-1.77; adjusted HR (aHR), 1.19; 95%CI, 0.80-1.76, compared to no switch). Patients switching to ABC-cART had a higher risk of VF (aHR, 1.99; 95%CI, 1.05-3.79) than patients not switching, patients switching to EFV (aHR, 0.82; 95%CI, 0.41-1.65) or NVP (aHR, 0.96; 95%CI, 0.44-2.07) having similar risk of VF compared to patients not switching.

## Conclusions

In previously ARV-naive patients, virologically successfully treated with a boosted PI-cART, switch to a NNRTI-cART, either EFV or NVP, is virologically safe, while switch to an ABC-cART should not be recommended.

